# Scaling and Sustaining Diabetes Self-Management Education in Community Pharmacies: A Statewide Collaborative Evaluation of Clinical and Operational Outcomes

**DOI:** 10.5888/pcd23.260258

**Published:** 2026-11-12

**Authors:** Christopher J. Daly, En-Ling Chen, Yewen Chen, Rachael A. Austin, Janice Kaelin-Kee, David M. Jacobs

**Affiliations:** 1 Department of Pharmacy Practice, Division of Outcomes and Practice Advancement, University at Buffalo School of Pharmacy and Pharmaceutical Sciences, Buffalo, New York; 2 New York State Department of Health, Albany, New York

## Abstract

**Purpose and Objectives:**

Community pharmacies are distinctively positioned to deliver diabetes self-management education and support (DSMES) to populations vulnerable to diabetes-related complications, prompting this evaluation of the program reach, clinical outcomes, patient-reported quality of life (QoL), implementation barriers, and cost efficiency of a statewide collaborative initiative in New York.

**Intervention Approach:**

The statewide DSMES initiative was designed to expand access to accredited DSMES services in community pharmacies and support long-term sustainability and included 1) pharmacy recruitment and accreditation, 2) a sustainability program, and 3) workforce innovation.

**Evaluation Methods:**

The mixed-methods evaluation included 44 accredited community pharmacies. Program reach was estimated using geospatial mapping and paired clinical and QoL outcomes were assessed through a continuous quality improvement framework. To evaluate a paraprofessional-supported hybrid workforce pilot, a comparative financial analysis was conducted using the 2024 Medicare Part B Physician Fee Schedule.

**Results:**

The initiative successfully reached priority populations, with 91% of participating pharmacies located in tracts that scored moderate to high on the Social Vulnerability Index. The program also yielded significant improvements in participant hemoglobin A_1c_ and median QoL scores. Additionally, it demonstrated that delegating administrative workflows to paraprofessionals reduced personnel costs from $6,592 to $2,169 per 6-patient cohort, transforming an estimated $4,318 net loss into a break-even financial model.

**Implications for Public Health:**

Integrating this paraprofessional workforce model provides a financially sustainable pathway for community pharmacies to deliver accessible and clinically effective DSMES to priority populations.

**Summary**

**What is already known on this topic?**

Diabetes self-management education and support (DSMES) improves clinical outcomes and self-care behaviors, but participation remains low and scalable sustainable delivery models are limited.

**What is added by this report?**

This evaluation demonstrated that a statewide community pharmacy–based DSMES model can expand access to DSMES in areas with high social vulnerability and can improve outcomes, including reductions in hemoglobin A_1c_ and enhancements in quality of life. The findings indicate that a sustainable approach through integration of a hybrid workforce model and billing infrastructure is feasible.

**What are the implications for public health practice?**

These results support community pharmacies as accessible and effective care settings for expanding delivery of DSMES and addressing disparities in diabetes care at the population level.

## Introduction

Diabetes is a major public health concern in the US; deaths and disability-adjusted life years associated with type 2 diabetes continue to increase (1). Global projections indicate that mortality rates due to type 2 diabetes will rise by 128.6% by 2050, disproportionately affecting members of racial and ethnic minority populations, people with low socioeconomic status, and those with lower educational attainment (2–4). In addition to increasing the likelihood of death, diabetes is associated with substantial illness, including cardiovascular disease, chronic kidney disease, and other long-term complications that contribute to reduced quality of life (QoL) and increased use of health care (5). Given these trends, scalable and sustainable strategies are needed to address diabetes at the health system level and reduce disparities in care.

Community pharmacies are distinctly positioned to reach priority populations. Patients visit their community pharmacist 1.5 to 2 times more frequently than their primary care physician, and most people live within 5 miles of a pharmacy (6). Pharmacists have expanded their role in delivering patient-centered services, including preventive care, screening, and management of chronic conditions, further establishing pharmacies as accessible points of care (7). Diabetes self-management education and support (DSMES) is an evidence-based program that empowers participants to manage their condition, resulting in decreased hemoglobin A_1c_ (HbA_1c_), improved medication adherence, and reduced risk of complications (8). National standards for diabetes education have been established since the 1980s; what is new about DSMES is that it is most commonly delivered within health system outpatient departments, specialized endocrinology clinics, or primary care settings. Capacity constraints, required travel, and long wait lists in these traditional settings often limit accessibility. Consequently, patient participation in DSMES remains low, with use rates among Medicare beneficiaries as low as 5.0% to 8.0%. Given their footprint in medically underserved areas and the high frequency of patient interactions, community pharmacies offer a highly accessible alternative setting to expand DSMES delivery. However, limited evidence on scalable and sustainable models for delivering DSMES in community pharmacy settings exists (9).

## Purpose and Objectives

From 2023 to 2025, a statewide DSMES initiative in New York was implemented and evaluated; data were collected throughout the implementation period. This initiative was conducted with the guidance of the Centers for Disease Control and Prevention (CDC) to expand DSMES access through partnerships with community pharmacies (10). The purpose of this initiative was to increase and sustain DSMES delivery within community pharmacy networks to improve program reach to priority populations. This program evaluation examined program reach, clinical outcomes and patient-reported outcomes (ie, QoL), implementation barriers, and cost efficiency of DSMES delivery in community pharmacy settings. Through a collaborative partnership between the University at Buffalo, the Community Pharmacy Enhanced Services Network of New York (CPESN NY), and the New York State Department of Health, the program aimed to establish pharmacy networks capable of delivering and billing for DSMES, assessing operational feasibility, and implementing workforce development strategies to support long-term sustainability across participating community pharmacies and their patients.

The primary evaluation goal of this initiative was to assess the implementation, reach, and cost efficiency of a statewide, community pharmacy–based DSMES program. Objectives were to 1) assess program reach across participating pharmacies and patient populations, 2) assess changes in clinical outcomes and patient-reported outcomes (ie, QoL), 3) identify implementation barriers, and 4) estimate personnel costs and cost efficiency to inform program sustainability.

## Intervention Approach

The statewide DSMES initiative was designed to expand access to accredited DSMES services in community pharmacies and support long-term sustainability. The intervention included 3 components: 1) pharmacy recruitment and accreditation, 2) a sustainability program, and 3) workforce innovation.

### Pharmacy recruitment and accreditation

Community pharmacies were recruited by CPESN NY based on their capacity, interest, location, and ability to reach priority populations (eg, Medicare beneficiaries and socially vulnerable areas). CPESN NY is a clinically integrated network of 287 community pharmacies throughout New York State providing patient care services to patients at high risk for chronic disease complications and adverse health outcomes (11). Participating pharmacies pursued DSMES accreditation through the American Diabetes Association or Association of Diabetes Care and Education Specialists pathways (12). CPESN NY provided centralized support and active coaching throughout the intervention. This technical assistance included establishing a cohort structure with an application timeline, policy and procedure manuals aligning with DSMES standards, in-person and virtual training to support the submission of applications, including the creation of patient charts required for accreditation, patient chart review, continuous quality improvement (CQI) implementation, and application preparation, as well as establishing ongoing office hours to support pharmacy staff. Pharmacies used a standardized DSMES referral workflow developed in coordination with CPESN NY that integrated point-of-care HbA_1c_ testing and identified prospective participants through various methods, including targeted review of dispensing data, in-store marketing, and local outreach. This standardized workflow also required the re-engagement of participants within 3 months of initiation.

### Sustainability program

To support ongoing program delivery, pharmacies participated in CPESN NY’s sustainability program for the duration of the grant period, which was established to support and maintain DSMES programs in community pharmacies. CPESN NY provided ongoing training, technical assistance, and support to programs on 3 main components: 1) CQI, 2) quality assurance, and 3) medical billing. Pharmacies were invited to participate in a more robust medical billing initiative to increase billing efficiency, provide regular feedback on claim status, and facilitate claim payment.

### Workforce innovation

To address staffing challenges, the initiative included a workforce innovation pilot involving top-of-license service delivery. DSMES program champions can include pharmacy technicians and interns. In the pilot program, 8 paraprofessionals, including pharmacy technicians and interns, managed administrative tasks of the program such as patient recruitment, onboarding, class coordination, and medical billing over a 6-month period. These 8 paraprofessionals were the DSMES program champions. The program champions completed a comprehensive training program in preparation for taking on the administrative tasks. A top-of-license delivery framework involves the pharmacist dedicating their time exclusively to providing clinical education while trained paraprofessionals assumed responsibility for the administrative and operational tasks. Using this framework was meant to shift operational tasks away from pharmacists and improve program efficiency.

## Evaluation Methods 

The evaluation used a mixed-methods approach to assess program outcomes and implementation. Evaluation measures were aligned with the study objectives. To assess program reach across New York State, we used geospatial mapping to identify participating pharmacy locations and link them to the CDC Social Vulnerability Index (SVI) at the census tract level. We measured patient engagement based on DSMES initiation (attendance at ≥1 session) and program completion.

For clinical outcomes and QoL, pharmacies used a CQI framework to collect paired metrics, including HbA_1c_, body mass index (BMI, calculated as weight in kg divided by height in m^2^), and patient-reported QoL, at baseline (Day 0, within 3 months of program initiation) and follow-up (Day 180, approximately 6 months post participation). Data were obtained via pharmacy point-of-care testing, health information exchange access, or medical provider reports. Paired comparisons were used to assess changes in clinical and QoL between baseline and follow-up. The normality of continuous data differences was assessed using the Shapiro–Wilk test, which indicated nonnormal distributions. Consequently, all clinical outcomes (HbA_1c_, systolic blood pressure, diastolic blood pressure, and BMI) and ordinal patient-reported QoL Likert scales were analyzed using the nonparametric Wilcoxon signed-rank test to determine statistical significance. Descriptive statistics for these paired outcomes are reported as medians and IQRs to accurately reflect the data distribution. Data analysis was conducted by using Microsoft Excel for Microsoft 365 Enterprise (Microsoft Corporation) and SAS version 9.4 (SAS Institute Inc). Significance was assessed by using a 2-sided *P* value of less than .05.

To identify implementation barriers, 2 quality assurance surveys were administered to participating pharmacies in August 2024 and March 2025. The pharmacies reported operational barriers, staffing capacity, workflow integration, and medical billing challenges.

To estimate personnel costs and cost efficiency, pharmacies reported approximate time required for DSMES-related tasks. These time estimates encompassed the entire DSMES workflow, including administrative tasks such as patient recruitment, onboarding, class preparation, postclass administration, and medical billing, and clinical tasks such as direct patient education. Pharmacies reported average staff time for these tasks, which was combined with role-specific salary data to extrapolate the total personnel costs for a standardized theoretical scenario of 1-hour DSMES classes delivered to a simulated cohort of 6 participants (reflecting standard Medicare Part B group billing models). Wage assumptions were explicitly derived from 2023 Bureau of Labor Statistics data for New York State (13,14), using average hourly rates of $63.96 for pharmacists and $20.83 for pharmacy technicians. Finally, these extrapolated personnel costs were compared against maximum potential revenue calculated by using the 2024 Medicare Part B Physician Fee Schedule (15) for upstate New York to evaluate the financial viability of a traditional pharmacist-only delivery model versus a paraprofessional-supported hybrid model. The University at Buffalo Institutional Review Board (IRB) approved the study (IRB STUDY5509), and this evaluation was reported in accordance with the SQUIRE (Standards for Quality Improvement Reporting Excellence) 2.0 guidelines (16).

## Results

### Program reach

During the grant period, 44 pharmacies achieved Association of Diabetes Care and Education Specialists or American Diabetes Association accreditation (Figure). Participating pharmacies were distributed across a range of social vulnerability contexts, deliberately targeting underserved populations. Based on the CDC SVI, most accredited sites (65.9%; n = 29) were located in high-SVI census tracts. An additional 25.0% (n = 11) were located in moderate-high SVI areas, whereas 6.8% (n = 3) and 2.3% (n = 1) were located in low-moderate and low SVI tracts, respectively. Across the network, 137 participants were onboarded; 98 participants (72.0%) attended at least 1 session, and 66 participants (48.0% of total onboarded participants) completed the full curriculum (defined as the comprehensive 10-hour educational series). The completion rate was 67.3% (66 of 98) among participants who initiated the program.

**Figure:**
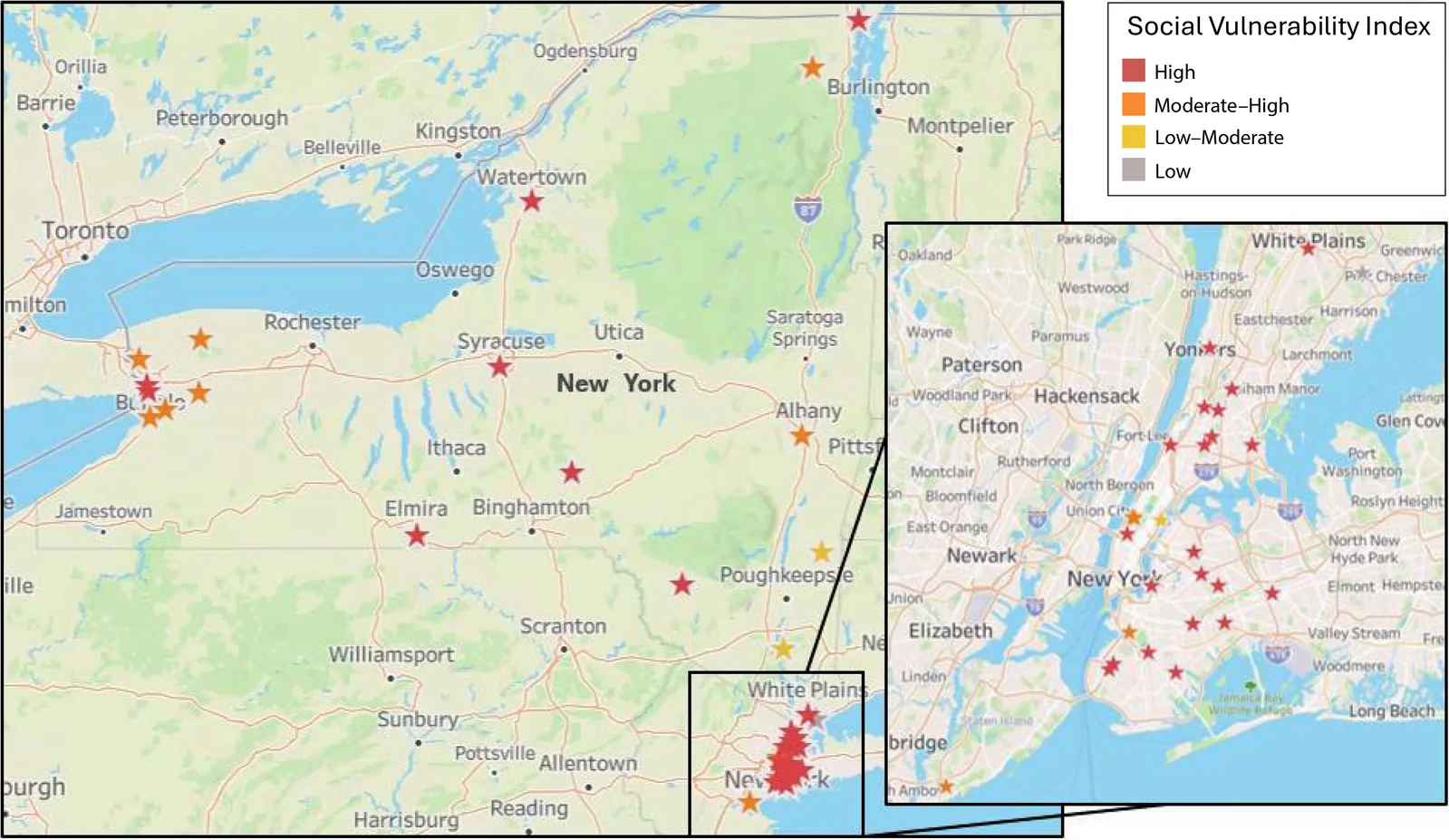
Diabetes self-management education and support program accreditation and geographic distribution by the Centers for Disease Control and Prevention’s Social Vulnerability Index (SVI) as of summer 2025 (N = 44). A map of New York that includes an inset of the New York City metropolitan area, displays the geographic distribution of 44 accredited community pharmacies. The pharmacy locations are marked to show their Centers for Disease Control and Prevention SVI category, indicating that most participating sites were concentrated in high and moderate-high vulnerability areas. Source: SVI Data & Documentation Download, Place and Health — Geospatial Research, Analysis, and Services Program (GRASP), Agency for Toxic Substances and Disease Registry (https://atsdr.cdc.gov/place-health/php/svi/svi-data-documentation-download.html).

### Clinical and patient-reported outcomes

We analyzed CQI metrics on a subset of participants (n = 61) who completed the program and had available paired baseline and follow-up data (Table 1). Among this subset, the data indicated significant improvements in participant health outcomes. In Year 1, 31 participants initiated the baseline CQI process, of whom 9 were excluded from the final analysis due to missing follow-up data, resulting in a final analytical cohort of 22 participants. This group consisted of an independent cohort of participants not eligible for Year 2 enrollment. Participants experienced significant reductions in HbA_1c_, with a median decrease of 7.2% to 6.6% (*P* = .002), and a significant decrease in BMI from a median of 31.5 to 31.0 (*P* = .02). Participants also reported significant improvements in their QoL, with median scores increasing from 3 at baseline to 4 at follow-up (*P* = .001). In Year 2, data collection confirmed continued clinical success among an additional distinct cohort of 39 participants, with significant improvements in both median HbA_1c_, which decreased from 7.9% to 7.6% (*P* = .002), and median QoL scores, which increased from 4 to 5 (*P* = .006).

**Table 1:** Baseline Demographic Characteristics of the Continuous Quality Improvement Patient Cohorts in a Statewide Community Pharmacy–Based DSMES Model, New York State

Characteristic	Year 1 continuous quality improvement cohort (N = 31)^a^^,^^b^	Year 2 continuous quality improvement cohort (N = 39)^a^^,^^c^
Age, mean, y	72	65
**Sex, no. (%)**		
Female	19 (61.3)	23 (59.0)
Male	12 (38.7)	16 (41.0)
**Race and ethnicity, no. (%)**		
Asian American	0 (0)	20 (51.3)
Black or African American	2 (6.5)	1 (2.6)
Hispanic or Latin American	2 (6.5)	3 (7.7)
White	27 (87.1)	14 (35.9)
Multiracial	0 (0)	1 (2.6)

Abbreviation: DSMES, diabetes self-management education and support.

a Demographic data were collected via the continuous quality improvement process, not standard pharmacy onboarding. Health insurance status was not collected within this framework.

b Year 1 demographic data was collected at baseline (Day 0); however, complete paired clinical outcomes were only available for 22 of these participants at follow-up due to attrition.

c Year 2 demographic data were collected at follow-up (Day 180), representing the final cohort with paired clinical data.

### Implementation barriers and cost efficiency

Results from the second quality assurance survey (88.0% response rate) identified the primary barriers to program sustainability. Pharmacies most frequently cited a lack of pharmacist staffing dedicated to the DSMES educator role and associated time constraints required to manage both the clinical and administrative tasks of the program (54%), as well as challenges with medical billing for DSMES services (54%) (Table 2).

**Table 2:** Top Pharmacy-Reported Barriers to DSMES Implementation and Comparative Income Versus Cost Analysis in a Statewide Community Pharmacy–Based DSMES Model, New York State

Top pharmacy-reported barriers to implementation and sustainability	No. of pharmacies (%), N = 37
Lack of pharmacist staffing dedicated to the DSMES educator role and time constraints	20 (54)
Medical billing related to insurance reimbursement for DSMES services	20 (54)
Lack of support staffing and time constraints	16 (43)
Patient attendance and retention	16 (43)
Integration of DSMES with dispensing workflow	12 (32)
Medical billing procedural and operational processes	11 (30)

Abbreviation: DSMES, diabetes self-management education and support.

To address the staffing barrier and evaluate financial sustainability, we conducted a comparative income versus cost analysis. We derived personnel costs from time estimates reported by a subset of participating CPESN NY pharmacies (n = 22). We calculated revenue using 2024 Medicare Physician Fee Schedule rates for upstate New York, assuming a standard 6-patient cohort where each patient completes the full 10 hours of initial DSMES (1 hour of 1:1 education [G0108] at $105.46/hour, and 9 hours of group education [G0109] at $30.40/hour). Under this fee schedule, the maximum Medicare Part B reimbursement for the 6-patient cohort is $2,274.36.

Based on the reported time estimates (n = 22), a traditional, fully pharmacist-led delivery model for this standardized cohort required substantial administrative time (averaging 25 minutes per patient for recruitment, 24 minutes for onboarding, 35 minutes per class for preparation, 24 minutes for postclass administration, and 24 minutes for medical billing), resulting in an estimated personnel cost of $6,592.14 (assuming a mean pharmacist wage of $63.96/hour). Against Medicare reimbursement rates, a pharmacist-led DSMES program would operate at an unsustainable net loss of $4,317.78 per 6-patient cohort.

To create a viable financial pathway, the program evaluated a hybrid top-of-license model. In this model, the pharmacist handles only the core clinical teaching (averaging 65 minutes per class), while a trained paraprofessional, the DSMES program champion, assumes responsibility for all operational tasks. By shifting these administrative tasks to the program champion (who in our study demonstrated time efficiencies averaging 22 minutes per patient for recruitment, 20 minutes for onboarding, 24 minutes per class for preparation, 19 minutes for postclass administration, and 18 minutes for medical billing; at a mean technician wage of $20.83/hour), total personnel costs for the cohort decreased to $2,169.06. This model reduced personnel costs by $4,423.08 per cohort, transforming the program from a substantial net loss into a financially sustainable service capable of breaking even (net profit of $105.30) against standard Medicare Part B rates (Table 3). This model inherently supports scalability. Because the pharmacist’s core teaching time is a fixed cost per class rather than a variable cost per patient, increasing the cohort size beyond 6 participants makes the model more cost efficient. As administrative tasks are completed by lower-cost paraprofessionals, the pharmacy can transition from breaking even into achieving a sustainable profit margin.

**Table 3:** Comparative Income Versus Personnel Cost Analysis for Standardized DSMES Delivery in a Community Pharmacy–Based Model, New York State

Income vs personnel cost for standardized delivery (n = 22)^a^^,^^b^	Pharmacist-only model, total cost, $^c^	Hybrid top-of-license model, role and total cost, $^d^
Clinical tasks^e^	692.90	Pharmacist, 692.90
Administrative tasks^f^	5,899.24	Paraprofessional, 1,476.16
Total estimated personnel cost	6,592.14	2,169.06
Estimated Medicare Part B revenue^g^	2,274.36	2,274.36
Net profit/(loss)	(4,317.78)	$105.30

Abbreviation: DSMES, diabetes self-management education and support.

a Scenario reflects a standardized delivery model of 10 1-hour DSMES classes delivered to 6 participants.

b Exact task durations used for these calculations vary by role. Total task times were calculated using multipliers factoring occurrences per class and per patient: tasks occurring once per participant (recruitment, onboarding) used a multiplier of 6; tasks occurring once per class (clinical education) used a multiplier of 10; and continuous tasks (class preparation, postclass administration, medical billing) used a multiplier of 60 (10 classes × 6 participants).

c Calculated assuming a single pharmacist performs all administrative and clinical tasks at a salary rate of $63.96 per hour, derived from 2023 Bureau of Labor Statistics data for New York (13,14).

d Calculated using a pharmacist salary rate of $63.96 per hour for core education and a technician salary rate of $20.83 per hour for all operational and administrative tasks, derived from 2023 Bureau of Labor Statistics data for New York.

e Clinical tasks include DSMES education.

f Administrative tasks include patient recruitment, onboarding, class preparation, postclass administrative tasks, and medical billing.

g Revenue was calculated using 2024 Medicare Physician Fee Schedule rates for upstate New York (15), assuming each of the 6 participants completed 1 hour of 1:1 education (G0108 at $105.46/hour) and 9 hours of group education (G0109 at $30.40/hour).

### Billing sustainability

Despite reported time-consuming billing challenges, pharmacies demonstrated an ability to generate revenue. Participating sites submitted 46 DSMES claims, of which 39 (84.8%) were successfully reimbursed during the 2024–2025 grant year. Participating sites secured payments across Medicare Part B, Medicare Advantage, and commercial payers, ranging from $35 to $150 per claim. Most claim denials were due to process-related errors (eg, nonrecognition of pharmacy provider status) rather than a lack of patient coverage.

## Implications for Public Health

Our evaluation showed benefits of sustaining DSMES delivery programs through affiliated community pharmacies. A central success of this public health–pharmacy partnership was its penetration into high-need areas. With nearly 91.0% of participating pharmacies located in moderate-high or high SVI census tracts, the program directly served patients at the greatest risk for diabetes complications (16). By situating accredited DSMES programs in neighborhoods with community pharmacies, the pharmacy-led model may help bridge critical gaps in chronic disease care for populations that traditionally face severe access barriers to health system–affiliated diabetes programs. Through a robust partnership with the state health department, CPESN NY established 44 new DSMES programs across the state and in areas with high social vulnerability and reached a diverse patient population through DSMES program delivery. Before implementation of this statewide community pharmacy–based DSMES model, only 2 pharmacies delivered DSMES in New York State.

Major barriers to program sustainability, including staff time constraints and medical billing, were addressed through CPESN NY’s sustainability program (17). Participation in the medical billing network allowed for monthly claim monitoring and feedback to be integrated into billing workflows, reducing claim submission errors, and facilitating paid claims. The DSMES program championed pilot-trained paraprofessionals on all aspects of DSMES operations, mitigating educator staffing shortages and time constraints, which together were the top reported barriers. This delegation of DSMES operations to paraprofessionals instead of pharmacists led to enhanced workflow sustainability without compromising clinical outcomes, a finding that aligns with existing literature on integration of paraprofessionals (18,19). Given the economic burden of treating diabetes complications compared with the substantial costs of treating long-term diabetes complications, an upfront investment in scalable community pharmacy DSMES programs is cost efficient. Furthermore, adopting this paraprofessional-supported model improves the financial viability of DSMES programs for community pharmacies, ensuring long-term program sustainability.

### Strengths and limitations

A primary strength of our evaluation is its real-world implementation across a diverse statewide network of pharmacies, demonstrating the practical feasibility of reaching vulnerable communities. However, our evaluation also had several limitations. First, participation and outcome data were not available from all accredited program sites, and participation data were self-reported. However, the primary purpose of this evaluation was to assess the operational feasibility and implementation of a community pharmacy model, rather than to re-establish the clinical efficacy of DSMES. Although our clinical data represented a subset of participants, the significant improvements in HbA_1c_ and patient-reported QoL observed in this cohort are consistent with past empirical studies indicating the extensive clinical benefits of DSMES (8). Additionally, the patient cohort was disproportionately Asian American (51%), a demographic skew driven by high program enrollment at specific participating pharmacies in the New York City metropolitan area. Due to this unique demographic composition and the focus on a single state, findings may not be generalizable to other geographic regions, broader populations, or different pharmacy networks. Our financial analysis has limitations because personnel costs were derived from self-reported time estimates and regional wage averages that may not fully account for variance in pharmacy overhead expenses. Furthermore, the revenue model assumed an ideal scenario of full patient completion under Medicare Part B fee schedules, whereas real-world profitability would fluctuate based on actual patient attrition rates and reimbursement variations across commercial and Medicaid payers. Patient recruitment barriers, coupled with the logistical challenges of obtaining long-term clinical metrics in a community setting, led to low numbers of participants with complete paired outcome data. Consequently, our clinical findings represent a subset of the total enrolled population; the missing follow-up data may introduce attrition bias and limit the ability to generalize the clinical effectiveness outcomes to the entire cohort. Furthermore, individual-level payer data (eg, distinguishing between Medicare and Medicaid beneficiaries) was not captured within the CQI framework, limiting our ability to stratify clinical outcomes or exact financial modeling by specific insurance types. Additionally, because data were collected through routine pharmacy workflows rather than a controlled clinical trial environment, we did not track whether participants were concurrently enrolled in other diabetes education programs (eg, those led by primary care, community organizations, or health systems). Simultaneous participation in other diabetes education programs or external support initiatives could confound our clinical findings, making it difficult to attribute the observed improvements in HbA_1c_ and QoL solely to the pharmacy-led intervention. Additional work is needed to address system-level barriers that affect patient referral and enrollment.

### Conclusion

Our model demonstrates the importance of community pharmacist-led support across the DSMES continuum in establishing and sustaining evidence-based diabetes care in community pharmacies and reaching populations vulnerable to diabetes-related complications. Specifically, the initiative successfully expanded access in high-need areas, yielded significant improvements in participant clinical and patient-reported outcomes, and proved financially viable when using a paraprofessional-supported workforce. CPESN NY and the New York State Department of Health continue to work together to support program enhancements, return on investment, and the reach of DSMES programs to New Yorkers at highest risk for diabetes complications and poor health outcomes.
